# Distinctive Delta and Theta Responses in Deductive and Probabilistic Reasoning

**DOI:** 10.1002/brb3.70179

**Published:** 2025-01-07

**Authors:** Emir Faruk Sevim, Yasin Yildirim, Esra Ünsal, Esra Dalmizrak, Bahar Güntekin

**Affiliations:** ^1^ Department of Neuroscience, Institute of Health Sciences Istanbul Medipol University Istanbul Turkey; ^2^ Research Institute for Health Sciences and Technologies (SABITA), Neuroscience Research Center, Clinical Electrophysiology, Neuroimaging and Neuromodulation Lab Istanbul Medipol University Istanbul Turkey; ^3^ Department of Physical Therapy and Rehabilitation, Institute of Health Sciences Istanbul Medipol University Istanbul Turkey; ^4^ Department of Physiotherapy and Rehabilitation Faculty of Health Sciences, Istanbul Gedik University Istanbul Turkey; ^5^ Department of Biophysics, School of Medicine Mersin University Mersin Turkey; ^6^ Department of Biophysics, School of Medicine Istanbul Medipol University Istanbul Turkey

**Keywords:** conditional reasoning, deductive reasoning, electroencephalography, event‐related oscillations, probabilistic reasoning

## Abstract

**Introduction:**

The neural substrates of reasoning, a cognitive ability we use constantly in daily life, are still unclear. Reasoning can be divided into two types according to how the inference process works and the certainty of the conclusions. In deductive reasoning, certain conclusions are drawn from premises by applying the rules of logic. On the other hand, in probabilistic reasoning, possible conclusions are drawn by interpreting the semantic content of arguments.

**Methods:**

We examined event‐related oscillations associated with deductive and probabilistic reasoning. To better represent the natural use of reasoning, we adopted a design that required participants to choose what type of reasoning they would use. Twenty healthy participants judged the truth values of alternative conclusion propositions following two premises while the EEG was being recorded. We then analyzed event‐related delta and theta power and phase‐locking induced under two different conditions.

**Results:**

We found that the reaction time was shorter and the accuracy rate was higher in deductive reasoning than in probabilistic reasoning. High delta and theta power in the temporoparietal, parietal, and occipital regions of the brain were observed in deductive reasoning. As for the probabilistic reasoning, prolonged delta response in the right hemisphere and high frontal theta phase‐locking were noted.

**Conclusion:**

Our results suggest that the electrophysiological signatures of the two types of reasoning have distinct characteristics. There are significant differences in the delta and theta responses that are associated with deductive and probabilistic reasoning. Although our findings suggest that deductive and probabilistic reasoning have different neural substrates, consistent with most of the studies in the literature, there is not yet enough evidence to make a comprehensive claim on the subject. There is a need to diversify the growing literature on deductive and probabilistic reasoning with different methods and experimental paradigms.

## Graphical abstract

There are significant differences in the event‐related delta and theta responses of deductive and probabilistic reasoning. In deductive reasoning, high delta and theta power were detected in posterior brain regions. A prolonged delta response in the right hemisphere and high frontal theta phase‐locking were detected in probabilistic reasoning.

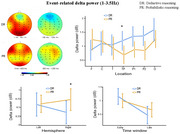

## 1 Introduction

To obtain new knowledge, we construct linkages between previously acquired knowledge. Deductive reasoning (DR) is reaching a conclusion by associating the premises we accept as true using formal logic rules. The syntactic structure of the premises leads us to the conclusion in this case. The conclusion is imposed on us by form. There is no room for ambiguity. If the rules are followed correctly, the conclusion is certainly true. The only case in which the conclusion would be false is if the logic rules were applied incorrectly (Oaksford and Chater 2007).

DR offers us a universe of truth with two options: true or false. We all know, however, that there is a considerably richer universe of possibilities in everyday life. The method through which we reach possible conclusions from our present knowledge is referred to as probabilistic reasoning (PR). In this case, there is no certainty provided by the rules of logic. However, we have the opportunity to talk on a probabilistic basis about situations where we cannot make a judgment within the limits of logic. The conclusion propositions provided to us by PR take on truth values ranging from false to true at varied levels of likelihood (Oaksford and Chater 2007).

Two different viewpoints contend to explain the neural foundation of reasoning. Mental logic theory highlights that DR operates through syntax and that language centers in the left hemisphere are responsible for DR (Rips 1994; Braine and O'Brien 1998). According to this idea, the logic rules are represented in the brain, and the relevant neural regions carry out the DR. However, this theory cannot tell us anything about PR because the logical rules do not lead us to a conclusion about PR. Mental model theory, on the other hand, claims that in reasoning, conclusions are reached by associating mental models of propositions (Johnson‐Laird 1994, 2010; Hinterecker et al. 2016). Our information is stored in the brain as mental models, and associating them can lead to certain and probable conclusions. Moreover, approaches that emphasize the pragmatic and probabilistic character of human reasoning in everyday life are becoming increasingly accepted. Based on Bayesian probability theory, this new paradigm defines human rationality in a broader framework and can explain situations considered as errors in logic (Oaksford and Chater 2007, 2020).

Many investigations have been carried out regarding the neural mechanisms of DR, with some producing contradictory results (Baggio et al. 2016; Reverberi et al. 2007; Bonnefond and Van Der Henst 2013; Noveck, Goel, and Smith 2004; Knauff et al. 2002; Rodríguez‐Gómez et al. 2018; Salto et al. 2021). Prado, Van Der Henst, and Noveck (2010) suggested that the contradictory findings in the literature are due to experimental paradigms that use different types of logical arguments, such as conditional and relational, even though they are all based on DR. Conditional reasoning is thus linked to syntactic processes, whereas relational reasoning is linked to visuospatial processes. According to a meta‐analysis, inconsistencies in the literature are not as prevalent as previously thought, and DR is conducted by a left frontoparietal network that may be separated into three groups based on the use of relational, categorical, and propositional arguments (Prado, Chadha, and Booth 2011). Another meta‐analysis study highlighted the sequential nature of DR; several brain regions are involved in this process, which has several stages (Wang et al. 2020). On the other hand, several studies on PR have been performed. Some studies focusing on information processing stages in PR have found activation in the prefrontal cortex, inferior parietal lobule, and occipital cortex (Liang et al. 2006; Demanuele et al. 2015). Uncertain PR tasks have been shown to induce activation in the right prefrontal cortex (Goel and Dolan 2000; Goel et al. 2007). However, the small number of studies on PR using different methods and paradigms is not sufficient to draw a holistic and general picture.

When we look at studies that directly compare the two types of reasoning, we again fail to find a consistent framework. While it is widely accepted that DR and PR are based on distinct neural substrates, there is disagreement over what these processes are and which brain regions they relate to. In some of the studies, DR is associated with language areas in the left hemisphere, whereas PR is associated with the left prefrontal cortex and, more commonly, the left frontal cortex (Goel et al. 1997; Goel and Dolan 2004). In contrast to these findings showing activation of the left hemisphere in DR, there are also studies showing that DR is predominantly associated with many different areas in the right hemisphere (Parsons and Osherson 2001; Osherson et al. 1998). Castañeda et al. (2023) found increased activation in the hippocampus during PR, which is compatible with the need to resort to everyday life information in PR. Some behavioral investigations also highlight that the neural systems used for DR and PR differ (Heit and Rotello 2010; Rotello and Heit 2009; Singmann and Klauer 2011). According to a meta‐analysis, DR is more important in verbal information processing, whereas PR is more critical in spatial information processing (Wertheim and Ragni 2018). Unlike all the aforementioned studies, Mansi et al. (2022) propose that DR and PR rely on numerous shared neural networks and that a single process performs the two types of reasoning.

To our knowledge, only one study compares DR with PR using EEG (Malaia, Tommerdahl, and McKee 2015). EEG recordings were acquired when healthy adults performed DR and PR, and the differences in event‐related potentials between these two types of reasoning were investigated. Malaia et al. (2015), unlike earlier research comparing DR and PR, presented the stimuli randomly rather than in blocks. Furthermore, the participants were not instructed on how to reason but instead had to make their own decisions within the task context. Responses were offered in four categories (certainly true, certainly false, probably true, and probably false), rather than two (true and false). As a result, when responding, participants had to choose both the reasoning type (DR or PR) and the truth value (true or false). It has been proposed that this design, which requires rapid switching between DR and PR, is more representative of real life. At the conclusion of the study, it was found that DR had shorter response times and that the N2 amplitude for positive and negative responses varied significantly in DR but not in PR. The findings were regarded as a partial separation of the neural processes that underpin these two types of reasoning.

There has been limited research into reasoning utilizing event‐related oscillations (Williams et al. 2019; Li, Sun, and Yang 2023). As far as we know, no study has been conducted to compare two types of reasoning in terms of event‐related oscillations. We believe that the event‐related oscillations approach has the potential to contribute significantly to understanding the neurophysiological underpinnings of reasoning.

The present study employed the event‐related oscillations approach to compare the neural processes underlying DR and PR. In designing our experimental paradigm, we followed the strategy proposed by Malaia, Tommerdahl, and McKee (2015), in which stimuli are presented randomly rather than in blocks, with four alternative answers. We agree with the perspective that this approach may represent the nature of reasoning in a way that is more compatible with real life. Because we represented reasoning types in our experimental paradigm similarly to Malaia et al. (2015), we expected reaction time to be shorter in DR, in line with their study. This hypothesis is also consistent with the idea that DR is a syntactic process with clear rules, in contrast to the relatively complex nature of PR. In both types of reasoning, we expected high delta and theta responses, which are likely to be involved in reasoning as they are associated with cognitive functions such as cognitive control, memory, and decision‐making (Güntekin and Başar 2016; Cavanagh and Frank 2014; Hsieh and Ranganath 2014). It was reasonable to expect this activity to spread in the frontal and parietal regions, often emphasized in the reasoning literature. In addition, we hypothesized that DR, which is usually associated with language areas, may show high activity in the left hemisphere. For PR, which is associated with spatial information processing, it was reasonable to expect high activity in the right hemisphere, but it was difficult to put forward a clear hypothesis since there are studies that point to different hemispheres.

## 2 Methods

### 2.1 Participants

A total of 20 healthy individuals, 11 of whom were female, all aged between 18 and 30 years (mean age (SD) = 22.8 (2.96)), and 16 of whom were right‐handed, participated in the study after providing informed consent and meeting the inclusion criteria. Our participants consisted of university students and community volunteers. Participation in the study was entirely voluntary, and participants did not receive any benefits. Only healthy individuals without any diagnosed neurological or psychiatric disorders were included in the study, while those who had used any substances that could affect cognitive functions were excluded. The study was approved by the Ethics Committee of Istanbul Medipol University (number of ethics: E‐10840098‐772.02‐2627).

### 2.2 Experimental Design and Procedure

**FIGURE 1:**
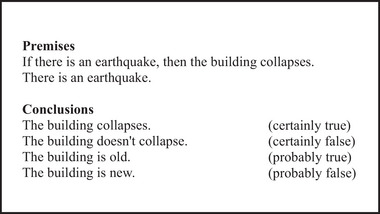
An example of an inference that was presented in the experiment. A premise pair followed by four alternative conclusion propositions is shown.

The stimuli consisting of inferences were prepared by using E‐Prime Software (Psychology Software Tools Inc., Pittsburgh, PA). The inferences used in the paradigm were formed on simple conditions based on daily life (Figure 1). All the premise pairs in the inferences were written in the modus ponens form (if P then Q; P). The truth value of the conclusion proposition (Q) required by the modus ponens form was given as certainly true. The opposite (not Q) corresponds to certainly false. Probable conclusions representing PR were written as statements that could not be given a truth value over the modus ponens form but were semantically related to the content of the argument. There was a total of 40 premise pairs that were followed by four alternative conclusion propositions with different truth values. Four alternative responses corresponded to the conclusions: certainly true, certainly false, probably true, and probably false. While “certain” answers represented DR, “probable” answers represented PR. An example of an inference is shown in Figure 1. The paradigm content was prepared by one of our colleagues trained in logic. Then another logician checked whether the inferences represented deductive and probabilistic reasoning. All stimuli were in Turkish.

**FIGURE 2:**
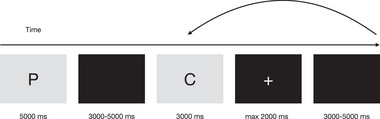
The experiment flow. After each premise pair (P), four alternative conclusion propositions (C) were randomly displayed. The back arrow indicates that other conclusions were shown before moving on to the new premise pair. Participants were instructed to respond when fixation plus appeared. The fixation plus disappeared when they responded, if they did not respond, it remained for a maximum of 2000 ms.

First, a premise pair appeared on the screen, and then the conclusions related to this pair followed. The order of presenting conclusions was randomized. Fixations (+) were placed after each conclusion for the participant to respond. Participants were asked to keep in mind the premises and to decide on the truth value of the alternative conclusions shown to them in writing on the screen. They had to decide not only whether each conclusion was true or false but also whether it was certain or probable. So, they chose which type of reasoning to use when deciding the truth value of a conclusion. When the fixation screen came, they had to press “1” for certainly false, “2” for probably false, “3” for probably true, and “4” for certainly true from the keyboard. The stimulus time for premise pairs was 5000 ms, and for each conclusion was 3000 ms. A fixation screen was displayed on the screen lasting 2000 ms. The interstimulus interval was added between each conclusion, varying randomly between 3000 and 5000 ms. The experiment flow is shown in Figure 2.

### 2.3 EEG Recording

EEG was recorded from Fp1, Fp2, F7, F3, Fz, F4, F8, Ft7, Fc3, Fcz, Fc4, Ft8, C3, Cz, C4, T7, T8, Tp7, Cp3, Cpz, Cp4, Tp8, P3, Pz, P4, P7, P8, O1, Oz, and O2 electrodes with the “BrainCap with Multitrodes” model cap (EasyCap GmbH, Germany) while participants were sitting in a dimly lit and shielded room. All electrodes were placed based on the international 10–20 system. References were two linked earlobe electrodes (A1 + A2). The electrooculogram was recorded at the left eye's medial upper and lateral orbital rim with Ag/AgCl electrodes. The impedance of all electrodes was provided below approximately 10 kΩ. The EEG was amplified using a BrainAmp MR plus 32‐channel DC system machine (Brain Products GmbH, Germany) with band limits of 0.01–250 Hz and digitized online with a sampling rate of 500 Hz.

### 2.4 EEG Analysis

The BrainVision Analyzer 2 software (Brain Products, Gilching, Germany) was used for data analysis. Independent component analysis was performed on the continuous raw data to remove artifacts due to eye movements. The moment the conclusion propositions appeared on the screen was considered the stimulus onset. The data was then divided into epochs according to stimulus onset. Epochs with incorrect answers or residual artifacts were eliminated by manual artifact rejection. Only correct answers were included in the analyses. The continuous wavelet transform was applied to each epoch for every subject and electrode for delta (1–3.5 Hz) and theta (4–7 Hz) frequency bands. A complex Morlet wavelet with 3 cycles was used for delta frequency between −3000 and +3000 ms and −1000 and +1000 ms for theta frequency for event‐related power and phase‐locking analyses. In the event‐related power analysis, the values were converted to the decibel (dB) scale as a normalization. In this dB normalization, prestimulus activity in the time window −500 to −200 ms was used as the reference interval in time (baseline). The same wavelet parameters were applied in the event‐related phase‐locking analysis, which evaluates how consistently the phase angle of signals aligns across trials. This type of analysis focuses solely on the phase characteristics of the signals, excluding any power features. Phase‐locking values range from 0 to 1, where values near zero indicate random phase angles, while values approaching one indicate strong phase alignment across trials. Finally, event‐related power and phase‐locking results were averaged across all participants.

### 2.5 Statistical Analysis

Statistical analyses were performed using Jamovi (The Jamovi project, 2021) and SPSS version 22 (IBM Corp., USA). Repeated measures ANOVA was applied to all the data. Behavioral data were analyzed using within‐subjects factors, including reasoning type (deductive and probabilistic). For the analysis of the event‐related power and phase‐locking, two reasoning types (deductive and probabilistic), two hemispheres (left and right), and seven locations (F3–F4, C3–C4, T7–T8, TP7–TP8, P3–P4, P7–P8, O1–O2) were included as within‐subjects factors. The event‐related power and phase‐locking analyses were performed separately in two time windows (0–600 ms and 600–1200 ms) for the delta frequency and one time window (0–300 ms) for the theta frequency, adding the aforementioned factors. For the comparison of reasoning type based on time window in the analysis of the delta frequency, reasoning type (deductive and probabilistic), two hemispheres (left and right), seven locations (F3–F4, C3–C4, T7–T8, TP7–TP8, P3–P4, P7–P8, O1–O2), and two‐time windows (early and late) were included for analysis as within‐subjects factors. Greenhouse–Geisser corrected *p* values were reported for the ANOVA analysis. Significant interactions were decomposed via follow‐up one‐way ANOVAs.

## 3 Results

### 3.1 Behavioral Results

**FIGURE 3:**
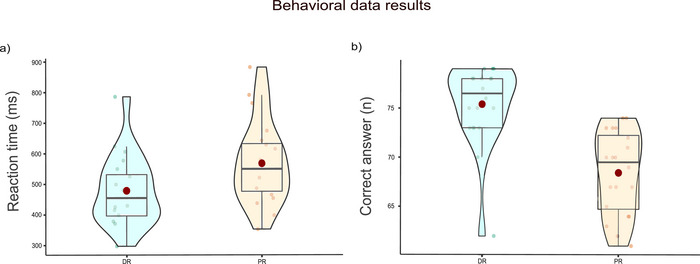
Behavioral data results. (a) The reaction time in DR (*M* = 479, SD = 25.3) and PR (*M* = 568, SD = 30.3), and (b) the number of correct answers in DR (*M* = 75.4, SD = 0.919) and PR (*M* = 68.4, SD = 0.939). Each small dot represents the values of the participants. Big red dots represent the mean value. Bold black lines in the middle of the boxes represent the median value.

A two‐way ANOVA was applied to reaction time and the number of correct answers given to stimuli. The reasoning type (deductive and probabilistic) was the factor. When we look at the results of reaction time, it was significantly higher in PR (*F*(1, 19) = 27.6, *p* < 0.001, $\eta_{p}^{2}$ = 0.593) than in DR. The number of correct answers given to DR out of a total of 80 answers was significantly higher than that given to PR (*F*(1, 19) = 118, *p* < 0.001, $\eta_{p}^{2}$ = 0.862) (Figure 3).

### 3.2 Event‐Related Power Results

#### 3.2.1 Event‐Related Delta Power Results

We used complex Morlet wavelet transform to obtain event‐related delta (1–3.5 Hz) oscillations in two different time‐frequency domains (early time window [0–600 ms] and late time window [600–1200 ms]) for all reasoning stimuli. A 2 (reasoning type: DR, PR) × 2 (hemisphere: left, right) × 7 (location: frontal [F], central [C], temporal [T], temporoparietal [TP], parietal1 [P1], parietal2 [P2], occipital [O]) repeated measures ANOVA with a Greenhouse–Geisser correction was applied to all data.

##### 3.2.1.1 Early Time Window

**FIGURE 4:**
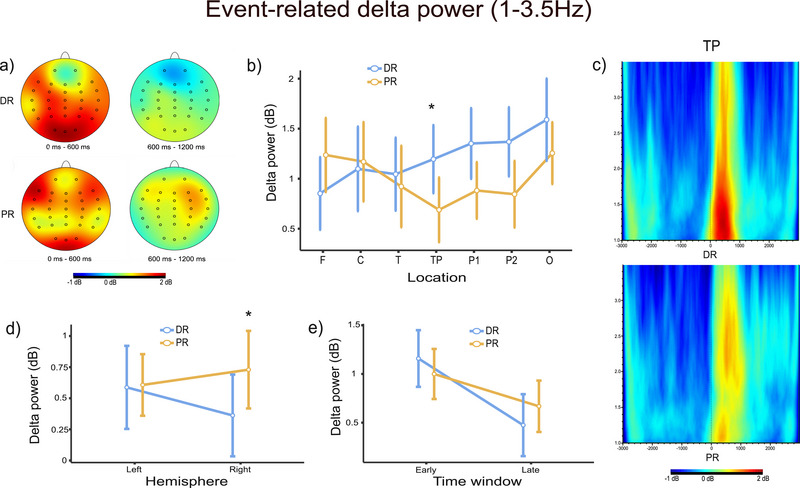
Event‐related delta power results. (a) Topographic distributions of DR (top) and PR (bottom) for early and late time windows. The red color indicates high power, and the blue color indicates low power. (b) Reasoning type and location interaction in the early time window (*p* < 0.01). (c) The grand average figures of event‐related delta power analysis (1–3.5 Hz) for TP locations in the time‐frequency domain during DR (top) and PR (bottom). Colors are coded in the same way as section A. (d) Reasoning type and hemisphere interaction in the late time window (*p* < 0.05). (e) Comparison of reasoning types in early and late time windows (*p* < 0.05). Asterisks indicate significant results.

The repeated measures ANOVA results showed that there was a significant difference in reasoning type and location interaction (*F*(3.39, 64.33) = 5.04, *p* = 0.002, $\eta_{p}^{2}$ = 0.21) (Figure 4b). After this two‐way interaction, separate one‐way repeated measures ANOVAs were performed for seven different locations to understand whether the expected reasoning type effect is location‐specific. Results showed that the reasoning type effect was significant in only one location (in TP: *F*(1, 19) = 4.62, *p* < 0.005, $\eta_{p}^{2}$ = 0.079). Accordingly, in TP, pairwise comparisons showed that participants had higher power for the DR (*M* = 1.136, SD = 0.172) than PR (*M* = 0.698, SD = 0.169). As seen in Figure 4b, although there is no significant difference in pairwise comparisons, it can be said that higher delta power is revealed also in parietal and occipital locations in DR.

##### 3.2.1.2 Late Time Window

The repeated measures ANOVA results showed that there was a significant difference between hemisphere and reasoning type interaction (*F*(1, 19) = 6.11, *p* = 0.023, $\eta_{p}^{2}$ = 0.009) (Figure 4d). Since we aimed to investigate the differences between the reasoning types, we applied new one‐way repeated measures ANOVAs comparing the reasoning type for each hemisphere. Our results were significant for only the right hemisphere (*F*(1, 19) = 7.52, *p* < 0.05, $\eta_{p}^{2}$ = 0.107). Accordingly, in the right hemisphere, the pairwise comparison showed that delta power is significantly higher for PR (*M* = 0.886, SD = 0.163) than DR (*M* = 0.348, SD = 0.192).

##### 3.2.1.3 Comparison of Reasoning Types Based on Time Windows

We applied another 2 (reasoning type: deductive, probabilistic) × 2 (hemisphere: left, right) × 7 (location: frontal [F], central [C], temporal [T], temporoparietal [TP], parietal1 [P1], parietal2 [P2], occipital [O]) × 2 (time window: 0–600 ms, 600–1200 ms) repeated measures ANOVA so that we want to investigate any differences between reasoning type based on the time window. As a result of that analysis, there was a significant difference between reasoning type and time window interaction (*F*(1, 19) = 9.43, *p *< 0.05, $\eta_{p}^{2}$ = 0.332) (Figure 4e). Since we aimed to investigate the differences between the reasoning types, we applied new one‐way repeated measures ANOVAs comparing the reasoning type for each time window. However, there were no significant differences between reasoning types for different time windows. When we compare the DR according to the time windows, there was a significant difference between the two different time windows (*F*(1, 19) = 20.6, *p* < 0.001, $\eta_{p}^{2}$ = 0.520). Early delta power (*M* = 1.237, SE = 0.163) is significantly higher than late delta power (*M* = 0.423, SE = 0.174). But there was no significant difference for PR's time windows (*F*(1, 19) = 3.31, *p* = 0.085, $\eta_{p}^{2}$ = 0.148).

#### 3.2.2 Event‐Related Theta Power Results

We used complex Morlet wavelet transform to obtain event‐related theta (4–7 Hz) oscillations in one time‐frequency domain (0–300 ms) for all reasoning stimuli. A 2 (reasoning type: DR, PR) × 2 (hemisphere: left, right) × 7 (location: frontal [F], central [C], temporal [T], temporoparietal [TP], parietal1 [P1], parietal2 [P2], occipital [O]) repeated measures ANOVA with a Greenhouse–Geisser correction was applied to all data.

**FIGURE 5:**
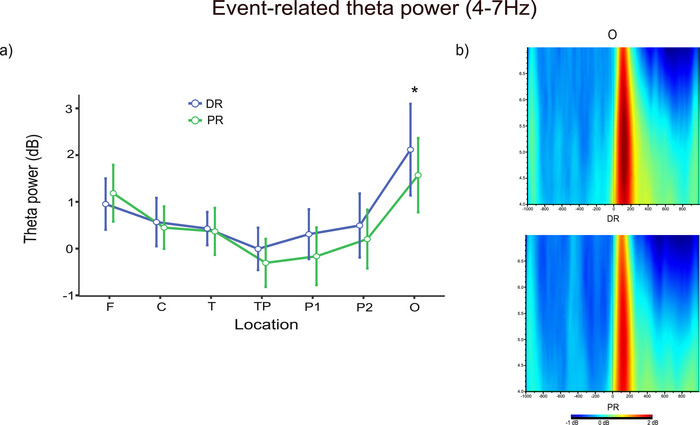
Event‐related theta power results. (a) Theta power results of reasoning type and location interaction (*p* < 0.05). (b) The grand average figures of event‐related theta power analysis (4–7 Hz) for O locations in the time‐frequency domain during DR (top) and PR (bottom). The red color indicates high power, and the blue color indicates low power. Asterisks indicate significant results.

The repeated measures ANOVA results showed that there was a significant difference between reasoning type and location interaction (*F*(3.01, 57.10) = 3.58, *p* = 0.019, $\eta_{p}^{2}$ = 0.008) (Figure 5a). To determine which reasoning type was specifically significant at which location, separate one‐way repeated measures ANOVAs comparing the reasoning type were applied for each location. Results showed that the difference between the reasoning types was significantly important for only occipital location (*F*(1, 19) = 5.81, *p* = 0.026, $\eta_{p}^{2}$ = 0.234). Accordingly, in O, event‐related theta power was higher for DR (*M* = 2.12, SD = 0.486) than PR (*M* = 1.57, SD = 0.379). Although not statistically significant, higher theta power in DR was also observed in temporoparietal and parietal locations (Figure 5a).

### 3.3 Event‐Related Phase‐Locking Results

#### 3.3.1 Event‐Related Delta Phase‐Locking Results

According to our repeated measures ANOVA results, there was no significant difference between reasoning type and location interaction (*F*(2.89, 51.94) = 2.477, *p *> 0.005, $\eta_{p}^{2}$ = 0.121) reasoning type and hemisphere interaction (*F*(1, 20) = 0.307, *p* > 0.005, $\eta_{p}^{2}$ = 0.017).

#### 3.3.2 Event‐Related Theta Phase‐Locking Results

Repeated measures ANOVA results showed that reasoning type and location interaction was statistically significant (*F*(2.24, 42.57) = 3.288, $\eta_{p}^{2}$ = 0.148). We applied other repeated measures ANOVAs to all locations to understand which type of reasoning was specific for which location. Our results were specific for the F location, PR (*M* = 0.474, SD = 0.024) had higher phase‐locking than DR (*M* = 0.415, SD = 0.027).

## 4 Discussion

Since not enough studies have been done on the subject, predicting which frequencies would be relevant for reasoning was difficult. According to Li, Sun, and Yang (2023), those with higher thinking dispositions had stronger frontal theta power in reasoning tasks. Similarly, Williams et al. (2019) reported increased frontal theta power in analytical reasoning. However, none of these studies provide a reliable prediction of the outcomes of a design comparing DR with PR. When we broaden our focus and look at the general theta literature, we see that memory, cognitive control, and working memory are associated with theta responses (Cavanagh and Frank 2014; Karakaş 2020; Hsieh and Ranganath 2014). Since these cognitive functions are very likely to play an important role in the execution of reasoning, we expected to observe high theta power, especially in the frontal region. On the other hand, delta responses have been associated with cognitive functions such as decision‐making, attention, and concentration (Güntekin and Başar 2016; Harmony 2013). It can be argued that these cognitive functions associated with delta responses are also significant in reasoning processes. Moreover, the N2 component is linked to delta and theta responses, which Malaia, Tommerdahl, and McKee (2015) found to be different between the two reasoning types (Harper, Knauff, and Johnson‐Laird 2014; Wienke et al. 2018; Hajihosseini and Holroyd 2013). That is why we focused on delta and theta responses in our research. We found that the reaction time is shorter, and the accuracy rate is higher in DR than in PR. DR produces more delta power in parietal, occipital, and particularly temporoparietal regions in the early time window. PR produces more delta power in the right hemisphere in the late time window. In the DR condition, delta power is significantly lower in the late time window than in the early time window, whereas in the PR condition, no significant difference is found between the time windows. DR produces more theta power in temporoparietal, parietal, and particularly occipital regions. Theta phase‐locking is higher in the frontal region in PR.

The fact that DR answers are more correct and reaction times are lower can indicate that DR is easier than PR. In DR, the process of applying logic rules to get a conclusion is more automatic. However, to overcome the PR task, a strategy based on prior experience and general world knowledge must be developed. Therefore, this was an expected result. Nonetheless, we cannot claim that PR tasks will always be more difficult than DR tasks. Different experimental paradigms may produce different results. Our findings agree with those of Malaia, Tommerdahl, and McKee (2015), who employed a similar paradigm. They also found that DR had a faster reaction time and a greater accuracy rate.

In the comparison of the time windows, from early to late in DR, there is a statistically significant diminish in delta power. The decrease in PR, on the other hand, is not statistically significant. The delta response in PR, which increases in the early time window, maintains its level to some extent in the late time window. The prolonged delta response in PR can be interpreted as a longer duration of mental effort required. This is most likely because PR tasks are more challenging, which is consistent with the behavioral data. Simultaneously, interpreting semantic content in PR may be claimed to require a different and more complex brain activation than in DR.

Both types of reasoning exhibited frontal and parietooccipital activity. This widespread activation is most likely related to several stages of the reasoning process. Frontal and parietal regions have previously been shown to be engaged in distinct phases of reasoning (Rodriguez‐Moreno and Hirsch 2009; Wang et al. 2020). In comparison to PR, posterior regions are coming to the fore in DR. In both the delta and theta bands, higher power was recorded in DR in posterior locations. This finding suggests that DR is more related to perceptual processes. The fact that the reaction time was shorter and the correct response rate was higher in DR also supports this. Participants responded quickly and easily in the DR condition.

In the reasoning literature, there are studies pointing to many different brain regions. For instance, research has indicated that engaging in DR triggers the activation of frontal areas (Goel et al. 1997; Goel and Dolan 2004). Our study's results share similarities with those of Castañeda et al. (2023), who found frontal and parietal (anterior cingulate cortex, inferior frontal cortex, and parietal regions) activity in DR. A separate investigation by Osherson et al. (1998) revealed the involvement of right parietal and occipital association areas in DR tasks. While we concur on the activation of posterior brain regions during DR, our findings diverge from this research when it comes to the comparison of brain hemispheres.

When examining the late delta responses to assess hemispheric distinctions, we observed elevated delta power in the right hemisphere during PR, which was an expected result. However, no significant difference in left hemisphere activation between DR and PR was evident. It can be inferred that the prolonged PR response is spread predominantly in the right hemisphere. Despite earlier research suggesting that the right hemisphere's significance in DR and the left hemisphere's in PR (Parsons and Osherson 2001; Osherson et al. 1998), a majority of DR studies highlight a frontoparietal network situated in the left hemisphere (Goel et al. 1997; Prado, Chadha, and Booth 2011). According to the mental logic theory, DR, which is based on language areas in the brain, can be expected to cause activation predominantly in the left hemisphere (Rips 1994). Conversely, the mental model theory, which views reasoning as a holistic process, emphasizes visuospatial areas and proposes the potential importance of the right hemisphere in reasoning (Johnson‐Laird 1994). In our investigation, the finding that PR elicits high delta power in the right hemisphere appears to align with the suggestion of the mental model theory. At least for PR, it seems plausible that the right visuospatial areas are involved in reasoning. Nevertheless, this cannot be generalized to the case of DR.

Previous research has documented theta phase‐locking during memory‐related functions (Klimesch et al. 2004; Rutishauser et al. 2010). PR involves a cognitive process where everyday information is utilized, mental imagery is engaged, and conclusions are reached by comparing known information. Given the nature of PR, it is reasonable to assert that memory occupies a crucial position in this cognitive process. Moreover, prior evidence has indicated the involvement of the hippocampus in PR tasks (Castañeda et al. 2023). However, theta phase‐locking has also been shown to be related to cognitive control (Cavanagh and Frank 2014). In their review of cognitive control, Mushtaq, Bland, and Schaefer (2011) emphasized the importance of cognitive control in adapting to uncertain situations. Therefore, the uncertainty in the PR condition may have led to the involvement of cognitive control mechanisms. We suggest that the role of memory in this context and the cognitive control mechanisms involved may have contributed to the high frontal theta phase‐locking we observed during the PR condition. On the other hand, memory and cognitive control are likely to have a more limited role in the DR condition, where reaching conclusions is achievable through syntactic structures.

Finally, we should mention some limitations of the present study. In the experimental paradigm, reasoning types are distinguished at the stage of determining the truth value of alternative conclusion propositions. Since this stage does not fully encompass the operations of integrating premises and thus reaching a conclusion, the inferential character of the process cannot be said to be directly represented in the paradigm. Second, we have analyzed the delta and theta frequency bands and presented our findings separately. The activation of reasoning at low frequencies could be revealed to be a single phenomenon if a wider frequency range were defined and analyzed. Third, for the reasons mentioned above, we focused on the two low‐frequency bands that may be most important in reasoning. However, other frequencies not analyzed in this study are also important for a holistic picture of the electrophysiology of reasoning. Fourth, because there are so few event‐related oscillation studies on reasoning, there are no studies similar to ours to compare our findings with. For this reason, we discussed our findings in the context of reasoning studies using various methods and event‐related oscillation studies on cognitive functions likely to be involved in reasoning. As more event‐related oscillation studies on the subject are conducted, it will become clearer how reasoning is related to different frequencies.

## 5 Conclusion

To conclude, we can say that the neural mechanisms on which DR and PR are based differ. The difference between the two types of reasoning manifests itself in several ways. DR produces more delta and theta power in posterior regions than PR, and PR produces more delta power than DR in the right hemisphere. High frontal theta phase‐locking is observed in PR. There may be a role for memory and cognitive control processes that are more plausibly involved in the PR than in the DR. In DR, which operates faster, the rate of correct answers is also high. However, a prolonged delta response is seen in PR with a longer reaction time and a low correct response rate. We can say that the results of PR, which show a prolonged delta response in the right visuospatial areas, are compatible with the predictions of the mental model theory. However, we do not agree with the view that a single process carries out the two types of reasoning. As most of the studies in the literature have pointed out, DR and PR are cognitive processes with distinctive characteristics.

Nevertheless, we still cannot say that our knowledge about the subject is sufficient. In order to uncover the neural correlates of reasoning, more studies using various neuroimaging techniques are essential. In the case of EEG brain oscillations, studies examining different frequency bands could improve the literature on reasoning. It is also essential to diversify the experimental paradigms used to represent the rich spectrum of this cognitive ability that we use in many different ways in everyday life. For example, a paradigm that represents the distinction between types of reasoning at the premise level and thus focuses more on the inference process could provide a more direct measure of the neural signatures of reasoning. If such a study using different groups of premises for different types of reasoning adopts the strategy of randomly presenting its stimuli, it could achieve a more realistic representation of everyday life.

## Author Contributions

**Emir Faruk Sevim**: conceptualization, writing–original draft, writing–review and editing, formal analysis, investigation, methodology, visualization. **Yasin Yildirim**: formal analysis, investigation, methodology, visualization, writing–original draft. **Esra Ünsal**: formal analysis, investigation, methodology. **Esra Dalmizrak**: formal analysis, investigation. **Bahar Güntekin**: methodology, resources, supervision, writing–review and editing.

## Ethics Statement

The study was approved by the Ethics Committee of Istanbul Medipol University (number of ethics: E‐10840098‐772.02‐2627).

## Consent

Informed consent was received from all participants.

## Conflicts of Interest

The authors declare no conflicts of interest.

### Peer Review

The peer review history for this article is available at https://publons.com/publon/10.1002/brb3.70179.

## Data Availability

The data that support the findings of this study are available from the corresponding author upon reasonable request.
